# “He’ll come with some sugar.” A qualitative study exploring the drivers and consequences of schoolgirls transactional sex behaviours

**DOI:** 10.3389/frph.2024.1325038

**Published:** 2024-05-09

**Authors:** Yandé Thiaw, Elizabeth Nyothach, Garazi Zulaika, Anna Maria van Eijk, Eunice Fwaya, David Obor, Penelope Phillips-Howard, Linda Mason

**Affiliations:** ^1^ Department of Clinical Sciences, Liverpool School of Tropical Medicine, Liverpool, United Kingdom; ^2^ Centre for Global Health Research, Kenya Medical Research Institute (KEMRI), Kisian, Kenya

**Keywords:** qualitative, transactional sex, Kenya, adolescent girls, schoolboys, parents, teachers

## Abstract

**Intoduction:**

Transactional sex (TS) is common in areas of sub-Saharan Africa, motivated by reasons beyond financial support. Through this qualitative study we sought to understand the motivation driving TS among adolescent schoolgirls in rural western Kenya where rates are reportedly high. Identifying and understanding drivers within the local context is necessary for implementation of successful public health policy and programming to reduce the associated harms impacting health and wellbeing.

**Methods:**

To understand the drivers of sexual behaviors, individual views, and socio-cultural norms, we spoke with schoolgirls, male peers, parents and teachers. The three latter groups may influence, encourage, and shape girls' views and behaviors and thus contribute to the perpetuation of cultural and societal norms.

**Results:**

One hundred and ninety-nine participants took part across 20 FGDs; 8 comprised of schoolgirl groups, and 4 each of schoolboy, parent or teacher groups. Through thematic analysis, poverty emerged as the key driver of TS and a normative behaviour amongst secondary school girls. Subthemes including parental influence, need for menstrual pads, pressure from boda boda drivers, peer pressure, and blame were part of a complex relationship linking poverty with TS.

**Discussion:**

We conclude that whilst TS is perceived as inevitable, normal and acceptable it is not really a choice for many girls. Exploring ways to encourage communication between families, including around menstruation, may help enable girls to ask for help in acquiring essential items. In addition, education at a community level may shift social norms over time and decrease the prevalence of age-disparate TS among schoolgirls and older, wealthier men in the community.

## Introduction

Transactional sex (TS) is defined as “*non-commercial, non-marital sexual relationships motivated by the implicit assumption that sex will be exchanged for material support or other benefits”* (1). There are some dissimilarities to sex work although the boundaries between the two are not always clear. Those who engage in TS often identify themselves as significant others, potentially as a girlfriend, boyfriend, lover, or “*sponsee*” of those who offer them financial or material support in exchange for sexual acts (2, 3). These acts may range from a one-off or occasional encounter to a long-term relationship and there may be shared emotional intimacy, with exchange of goods or money being implicit and demonstrating commitment (4). In contrast, sex work describes the relationship between that of a client and provider, whereby the giving of money or gifts is explicit with little, if any, emotional intimacy (1).

TS rates range among adolescent girls and young women (AGYW) from 2.1% to 52% across SSA countries (1), indicating that a substantial proportion of AGYW in SSA participate in this activity. Between 36% and 80% of sexually active adolescent girls ages 12–19 reported ever having had TS in a 4-country study (5). Nearly a tenth (8.7%) of AGYW reported having practiced TS with a casual partner in rural South Africa (6), while another study in South Africa reported 10% of sexually active AGYW had participated in TS (7). In rural Kenya, 52% of sexually active girls aged 14–17 years reported having practiced TS (8), whilst in Tanzania 57% of sexually active secondary and university students reported having practiced TS with a “sugar daddy” (9). As engaging in TS is a socially stigmatized activity, these self reported statistics may have underestimated the true rates of TS among AGYWs.

It is assumed AGYW are susceptible to TS because they rely on gifts and money to meet their basic and material needs (10). However, motivations behind forming these relationships are complex and extend beyond AGYW's need for necessities. While many authors describe a traditional societal norm wherein a man is expected to provide for his partner and receive sex in return (11, 12), others suggest TS is proactively driven by the recipient seeking an opportunity to acquire “luxury items”, and convey a higher social status amongst peers or within in the wider community (13). Sexual agency or power is also cited as a motivator of TS (10), while others evidence that gift giving is a natural expression of respect within a loving relationship (14).

Understanding the context behind TS is critical to gauge the degree of choice within a sexual relationship and the potential for harm (impacting health and wellbeing amongst AGYWs) resulting from unequal power dynamics. TS is frequently associated with several risk behaviours that impact AGYW sexual and reproductive health, including multiple partners (15), age-disparate relationships (16), inconsistent condom use and sexual and/or physical abuse (17–19). Duby et al. (7) reported contradictory dynamics in that girls reported self-agency in choosing TS behaviours, but at the same time had little power to insist on condom use (7). Research indicates that TS - alongside biological susceptibility and general poor access to health care in the region - may be among the key behavioural practices contributing to the wide gender disparity in HIV among young people in sub-Saharan Africa (6). In Kenya, the prevalence of HIV infection is 3% among adolescent girls between the ages of 15–19 years old, four times higher than among boys the same age (20).

In Kenya, TS appears to be largely accepted in the community and a societal norm among AGYW (21, 22). One theory posited that children who have been orphaned in Kenya are more at risk of engaging in riskier sexual behaviours such as TS; however, Juma et al. (8), refuted this claim concluding that other factors such as funeral discos, parental inability to provide necessities, and unsupervised sleeping arrangements had a much greater impact on adolescent risk-taking behaviour when compared to TS in rural western Kenya (8).

Despite high reported prevalence (8), research focusing on TS among AGYW in Kenya still lacks key insights. There is a paucity of literature relating to the drivers and motivations of TS from those who have first-hand knowledge and experience of the situation. There is also a dearth of literature incorporating male perspectives. Understanding the complexities and drivers of TS in a specific context can inform more effective initiatives and can contribute toward reducing the negative consequences of TS. The purpose of this qualitative paper was to understand the motivation driving TS among adolescent schoolgirls, not only from their perspective but also from those of their male peers, their parents, and teachers who may influence, encourage, and shape girls’ views and behaviours, (and may participate as transactional partners), and reinforce cultural and societal norms. This may facilitate a deeper understanding of the drivers to girls beliefs and behaviours whilst also providing context to any matters of conflict arising throughout the narratives.

## Methodology

### The main trial study design

The data analysed in this paper were collected in a 4-arm cluster randomized controlled trial providing interventions to reduce sexual and reproductive harms and school dropout among adolescent schoolgirls between 2017 and 2021 [described in detail in Zulaika et al. (23)]. The interventions in the randomized controlled trial were: (1) a menstrual cup; (2) conditional cash transfers each school term; (3) a menstrual cup with termly conditional cash transfers; and (4) control - girls in this arm continued their regular menstrual management practices. The trial was conducted in 96 study area secondary day schools which were girls only or co-educational and gave approval to participate by the head teacher. Schoolgirls were eligible if they were resident of the area and non-boarding, had reached menarche, had no disability preventing participation and were not visibly pregnant. We targeted Form 2 girls for recruitment to maximize time under study. Additionally, they had to provide parental/guardian consent and give personal assent to participate. The median participant age was 17.1 years.

### Study setting

The study was conducted in Siaya County, western Kenya. The total area is approximately 2,500 km^2^ with a population of nearly one million (24). Siaya is a rural and impoverished area comprising mainly of the Luo ethnic population, the majority of whom are fisherfolk, subsistence farmers. Sexual and reproductive health is poor, with the highest prevalence of HIV (21%) among 15–64-year-olds nationally and an age specific fertility rate for girls 15–19 of 97 births per 1,000 (24). A recent study reported 23.3% of 13–19-year-old girls had ever been pregnant (25).

### Sampling and recruitment for focus group discussions (FGD)

FGDs were used to generate data because of their suitability for collecting information on communal views as well as their ability to collect data on group interactions and differing viewpoints within a community (26).

A random selection of a sub-set of 1 in 10 schools (with one class per school) was used to select each study school from which girls enrolled in the trial were invited using simple random sampling to attend FGDs (8 FGDs, 2 per intervention arm). Any girl participating in the main trial was deemed eligible for participation in an FGD. The eligibility requirements for boys, parents and teachers stemmed from relationship to the participating girls i.e., boys were eligible if they were in the same class as girls, parents were eligible if they had a daughter participating in the study, teachers were eligible if they taught at one of the schools selected in the main study. Boys were invited to participate during class meetings about the study. Parents of these pupils were invited when they provided consent for their daughter or son to participate in an FGD. Teachers' meetings were used to recruit teachers. The FGDs took place within schools, (or at a place in close proximity to the school for the parent FGDs), in a room where quietness and privacy were ensured. These were all conducted at baseline prior to intervention provision.

As the main focus of the study was girls, we held 8 FGD with these participants, and 4 FGD each respectively with boys, teachers and parents.

We aimed to recruit between 8 and 12 participants in each FGD (27, 28), however in school 10 all teachers wanted to participate, and we felt it was not appropriate to refuse a sole member of staff and thus continued with 13.

Each FGD utilized a semi-structured schedule which allowed us to focus on topics pertinent to our study aim the aim was to understand girls' pre-study menstrual practices, cash availability and use, and socio-behavioural characteristics including sexual risk behaviours. The core topics for each focus group category (i.e., schoolgirls, or schoolboys) differed slightly to accommodate the perspective shift in the participants. However, the main focus for all FGDs were as shown below (See Table 1). Conversations specifically surrounding transactional sex were however frequently initiated by participants and probed further by the moderator. e.g., when asking what menstrual products girls preferred to use, and following with how they obtained sanitary pads, numerous participants described girls receiving directly from sponsors/sugar daddies, or using monies obtained from sexual partners. Whilst not intending to be the focus of the qualitative investigation, the recurrence of this topic led us to conclude its' importance to the study background as well as to the lives of these participants. The data was thus plentiful to analyse in its own right as a stand-alone paper.

**Table 1:** FGD topics by group.

Topics	Schoolgirls	Schoolboys	Parents	Teachers
Warm up and overview	Yes	Yes	Yes	Yes
School absenteeism, drop-out and prevention	Yes	Yes	Yes	Yes
Experience and challenges around commitments at home	Yes	No	Yes	No
Experience and challenges around menstruation	Yes	*Boys perceptions around menstruation*	Yes	Yes
Experience and challenges around money	Yes	Yes	Yes	Yes
Experience and challenges around sex	Yes	*Boys sexual relationships with girls*	Yes	No
Experience and challenges around travel and leisure time	Yes	No	Yes	No

Each FGD was attended by an experienced moderator with a note taker alongside. Audio recordings were taken, and these were translated/back translated from Dholuo (if appropriate) and transcribed verbatim by either the moderator or note-taker. Both the moderator and note-taker were young Kenyan women local to the study area and proficient in Dholuo and English.

### Analysis

Following the framework designed by Braun and Clarke, thematic analysis was used to facilitate both a deductive and inductive approach to ensure our research questions were answered whilst also allowing topics important to our participants to emerge (29). Each transcript was read and re-read to gain familiarity, and a coding framework was created with codes then assigned to relevant portions of the transcripts. The codes were constructed into broader categories which were then merged into key themes as applicable. The researcher compared and contrasted codes and themes across and between schoolgirls, parents, teachers, and schoolboys' narratives. To minimize bias, two of the authors (LM, PPH) read each transcript, checked the coding frame, and discussed the resultant analysis to ensure the themes incorporated, and conclusions made represented as much as possible the key opinions given by study participants.

### Ethics

The trial received ethical approval from the Scientific and Ethical Review Boards of the Kenya Medical Research Institute (#3215) and the Ethics Committee of Liverpool School of Tropical Medicine (15-005). Schoolgirls and schoolboys provided written assent/consent dependent on age, whilst parents and teachers provided written consent prior to the discussion starting. This included agreement to having the discussion audiotaped.

## Results

Two-hundred participants took part in this study (Table 2). Eighty were schoolgirls and 35 schoolboys, between 15 and 18 years old. Thirty-nine parents, 35 women and just 4 men (one in each group) participated. Of the 46 teachers who took part, the majority were men with just 9 female participants (2 women in S9, S8, and S6, and 3 women in S2).

**Table 2:** Focus group participant demographics.

School	1	2	3	4	5	6	7	8	9
Schoolgirls									
Age range	16–17	16–18	17–18	15–17	15–17	15–18	16–18	15–17	
Ethnicity	All Luo	All Luo	All Luo	1 × Luhya	1 × Luhya	2 × Luhya	All Luo	All Luo	
Participants	10	9	12	7	9	11	11	11	
Length FGD minutes	56	68	81	55	85	60	78	38	
Schoolboys									
Age range		16–18			16–17	17–19		16–17	
Ethnicity		All Luo			All Luo	1 × Luyha		All Luo	
Participants		9			9	9		7	
Length FGD minutes		90			80	67		64	
Parents									
Age range		24–52			36–70	30–65		28–60	
Ethnicity		Luo			1 × Luhya	1 × Luhya		Luo	
Male/Female		1/8			1/6	1/9		2/10	
Participants		9			7	10		12	
Length FGD minutes		80			92	79		102	
Teachers									
Age range		20–50				21–53		26–31	24–53
Ethnicity		3 × Luhya				1 × Luhya		1 × Abujuba	All Luo
Male/Female		7/5				6/2		8/3	9/2
Participants		12				8		11	11
Length FGD minutes		67				110		125	60

Two main themes were identified through deductive analysis: (1) Drivers of TS and (2) Consequences of TS. Within the theme “Drivers of TS” several sub-themes emerged: poverty, parental influence, menstrual pads, boda boda drivers (motorbike/bicycle taxi operators), peer pressure, sexual pleasure, and blame. The subthemes arising from “Consequences of TS” were school dropout; pregnancy; and STIs.

## Drivers of transactional sex

### Poverty

TS was perceived as a normal part of the community among the discussants, with recognition that it was predominantly impoverished circumstances that pushed girls into it. This finding was evidenced consistently across most FGDs. Usually, TS was recognized as having to “*pay*” or “*pay back*” for monies or goods received.

“*You know most girls when they do not have pads, they will have to go seek for pads, and in the process, she may have sex with anybody so that she can be given money to buy pads, they will practice prostitution and they may have sex without protection” (P2, S5, Girls)*

The typical perception across all groups was that deprivation caused a gap in meeting girls' basic needs, including those of food security at times. Schoolgirls had little opportunity to earn money to meet their needs other than by engaging in TS. This was commonly acknowledged and usually stated as a matter of fact.

“*Some of them the problems they are having at home, makes them engage in sexual relationship, you will find at home they are poor, there is no way of getting food, like right now there is famine there is food shortage, so when she gets a chance with a motor bike operator who can give her money or supper hence having something to eat when she goes back home.” (P5, S8, Boys)*

*“The main undoing is poverty, most of them come from very poor background, and that is why you may blame the parents that they don't provide, but the parents cannot even afford the meal maybe one meal in a day, so sometimes we blame but we look at the poverty around and that is also a great influence.” (P1, S6, Teachers)*

There was recognition in girl and parent FGDs that some girls chose to have multiple partners because of the financial advantages that it bestowed.

“*They normally have many [men who they engage in transactional sex with], up to even five so you can ask all of them money and when almost all of them give you money, you will be fortunate.” (P1, S5, Girls)*

### Parental Influence

Narratives across all groups frequently described the lack of parental contribution toward their daughters' needs, recognising that parents themselves struggled to make ends meet. This invariably led to girls having to find their own ways to obtain their needs. Many parents themselves described their struggle to support their daughters, fully aware, and often fearful, of the consequences if they were unable to do so. Some mothers clearly did not want their daughters to resort to engaging in TS to get by. Some also told of circumstances in which they (or others) were unaware of the situations their daughters had got into because girls hid or were secretive about their behaviours.

“*But for a woman, if her daughter comes to her, I do not think if there is a woman who can do such a thing, I think I always compare with my own daughter, her father may respond to her carelessly, but I always try to get something to sell, maybe my beans or maize so that I can help her with what she needs. … Sometimes your daughter is in school or college and she needs some money, her father will just respond carelessly, ‘I do not have money’! She will then make a phone call crying, and as a mother you are forced to find out how you can help her, because if you don't, where will she get it, in other words you are telling her to get it from somewhere else.” (P4, S6, Parents)*

*“You know it is not easy for a girl to ask her father, but you as a parent it is upon you to provide for her because if you don't, we have shared that a time will come when someone else will start providing what you cannot give her.” (P10, S6, Parents)*

*You may find that sometimes you are not aware your daughter has started intimate relationships, and may be the person who is sleeping with her is a very old person. Maybe when you ask where she has been she will say, Mr. so wanted to see me, and if you ask why, she says, he wanted to ask me about school. So as a parent you won't imagine anything, maybe this old man is a very close relative, or is the intermediary in the relationship and is benefiting. Or it's her grandmother who has nurtured this relationship and is also benefiting. Because when this man comes to the grandmother's house, he'll come with some sugar.*” (P1, S5, Parents)

By contrast some parents, teachers and boys' narratives agreed with a number of the girls who told of parents or guardians who push their daughters towards TS by making it clear that they cannot, or will not, provide necessities for them. Some narratives described how parents turned a blind eye while others described parents being actively involved in pushing their daughter into a sexual relationship to obtain necessities. Sometimes this was just to ensure girls were self-sufficient in their own personal items, but other times benefitted the household. The following quotes illustrate the different nuances evident in their narratives.

“*…like one of them told me, that the grandma told her; you are now big enough to fend for yourself, you can't use your brain. So it means that this parent or the guardian is giving the girl a free hand in going to fetch for the money. The only way a girl can get money, the quickest way, probably now that she is needed in school and most of the time she cannot go to the farm and wait would be, especially to get somebody who will give her a handout.” (P2, S8, Teachers)*

*“I have seen a parent who when he noticed the child was doing such things, he just told the child, my dear tell that man to bring me sugar. So, I do not know why this man giving the parent sugar is of help to the child or the parent. So, I do not know what to say, I do not know if it's right to say they are selling their children.”—(P6, S5, Parents)*

*“By the way, this all depends with the parents. Someone like my aunt, if you stay with her, she will always want you be smart all the time. She wants you to wear make ups, and she buys them for you. And then when she gets someone with money she connects you. So you will be forced to pay that man a visit even if you do not feel like, you will have to because she wants you to go with him. You will have to go with that man whether you like it or not. Because she expects you to bring her a full box with shopping.” (P12, S3, Girls)*

Difficulties in communicating with parents about their needs was also recognized as a contributor to girls having to obtain necessities by their own means. Reasons cited for these difficulties included embarrassment in having to ask for personal items such as underwear or menstrual pads, exacerbated by girls' domestic arrangements, namely living only with male relatives or elderly grandparents. Relationships with parents or guardians were often strained; girls frequently accused parents of being “harsh.” Some narratives went further with participants of all types describing instances of parents or guardians not providing necessary items such as underwear or insisting that the adolescent girls in their charge be self-sufficient.

“*Like me I am staying with my father, how will you tell him you need panties?” (Laughter). (P5, S3, Girls)*

*“You are talking of your father! There are also such mothers! By the way, even my own mother, she tells me, you are now a big girl, you need to provide for yourself. And she says, when she was my age, her mother never provided for her. So you will be forced to go and get that money from someone who can give you, to buy those inner wears. Those panties, bras and bikers. (She giggles), imagine if I ask my mother she tells me, go and look for it, so what will you do, you will be forced to go and get from your boyfriend.” (P1, S3, Girls)*

### Menstrual pads

The need for menstrual pads was perceived by a few participants as the tipping point into TS.

“*Sometimes it is just the circumstance that pushes the girl, for instance, you do not have money to buy pads; and this is the major thing that pushes most girls to start sexual relationships. And what I am saying is very true.” (P5, S3, Girls)*

No other item was described in this way. The importance of TS as a means to obtain sanitary pads was further highlighted by the numerous references that participants echoed across all groups.

“*Pads is one of the basic necessity that these girls need and it becomes very shameful…it's embarrassing because some of them they are in a mixed school and so they feel like this is one of the things they should be having but then probably the parents cannot provide that.” (P2, S8, Teachers)*

*“Maybe your parents cannot afford it [menstrual products] due to poverty back at home, and then they [adolescent girls] get a boda boda…who is ready to offer her a packet of these sanitary pads, and you know, we do not have free things nowadays, in exchange maybe they may do those other things.” (P5, S8, Teachers)*

### Boda boda drivers and other sexual partners

The need for transport to and from school also emerged as a key driver of TS with boda boda (motorcycle taxi) operators blamed as a significant architect of TS. Frequently FGDs discussed schoolgirls’ needs for a lift from boda boda drivers which had to be reciprocated with sex. Girls themselves acknowledged that accepting a ride meant they became trapped into a relationship or one-off sexual encounter with the boda boda driver as payment. Girls spoke of being aware that when requesting a ride from a boda boda driver they would be asked for sex in lieu of payment.

“*When I go somewhere, the boda boda guy just told me, ‘Madam, come I give you lift!’ I accepted the lift because I was tired. So, on our way, he said, you are very beautiful! And then he continued, would you mind giving me your contacts. And then I told him, I do not have contacts. He then said, you are not telling the truth. I then just told him, I do not have contacts, but just get me to school, we will connect afterwards. And then he said, no way, if you do not have the contacts, I will take you back to where I found you … or you give me what I want.”—(P9, S3, Girls)*

*“Girls do walk when they are coming to school, but if your home is far, and the parent you live with cannot afford the transport, that will make you approach those guys for friendship because you know they will be giving you lift to school.”—(P9, S2, Girls)*

While narratives tended to focus on boda boda drivers as the primary predators, when questioned specifically about who paid girls for sex or presented them with gifts, participants described a range of actors from peers to “old” men, from blood relatives and extended family members to figures of authority. There was some recognition including among the schoolboys, that older men, often called sponsors, were able to offer greater financial rewards than younger men or classmates and so may be more favoured as transactional partners.

“*There is not any girl who can befriend you unless you are in a position to provide her with the needs, because she cannot tell you to buy her pads because even you, you still depend on your parent, so where will I get the money to buy for her.” (P3, S2, Boys)*

Even teachers could offer girls help with their schooling in return for sexual favours according to some teachers themselves and verified within our girl FGDs.

“*Even if you go to the teacher, the teacher may tell you he is busy come back another time. So, when you go another time, instead of him teaching you, he will have his own agenda. So, you know catching up is difficult, you will end up just failing.” (P9, S3, Girls)*

*“You mentioned that instead of the teacher helping you, he has his own agenda”? (M of S3 Girls)*

*“Yes. Maybe he wants to have sexual relationship with you. And then another thing, let's say the teacher is your boyfriend, you know when he comes to class you will not concentrate. You will only be thinking the things he was doing to you. So, you know we really have a problem here.” (P9, S3, Girls)*

*“Help me understand something; do girls have relationships with teachers”? (M of S3 Girls)*

*“Yes.” (All)*

*“Let's say the teacher is making advances on you, you as a student, you will accept so that when exams are done, he will steal for you, [grade inflation]. So that is where the trick is, you will depend on him to maintain the standards.” (P9, S3, Girls)*

*“Okay.” (M of S3 girls)*

*“You know when you are in a relationship with the teacher; you know your status is raised. If you have relationship with teacher, you feel you are at the top because he provides for you. They can even help you inform of education. But there are others who just use you.” (P3, S3, Girls)*

### Peer pressure

Aside from a lack of basic needs, the role of peer pressure was discussed in most FGDs and designated as an important driver of schoolgirls' engagement in TS by all participant groups. This reason for engaging in TS appeared less because necessity but more borne out of choice. The schoolgirl FGDs noted that many girls perceived there were benefits to TS because it gave them access to desirable items they otherwise would not be able to afford, that their friends and classmates had.

*“They engage because of the peer pressure, you know as they grow, they experience some emotional changes, so they will just have peer pressure which will lead them to having sexual relationships.” (P5, S2, Boys)*

*“The reason girls have boyfriends, is because of peer pressure. Let's say if someone has a boyfriend she will say: “Hey look, my boyfriend has bought me this and that.” So, any time I see her, she is smartly dressed, if I ask her, she says: ‘Onyango my boyfriend…’ so I will admire her life and say even me I have to get someone to also buy for me.” (P1, S2, Girls)*

*“There is a misconception that when a girl reaches a certain age, either a girl is in form three, and still not having a boyfriend, this girl will think that I am still lagging behind, because all the girls around me are having boyfriends and there is some good thing they are getting from that… So they believe that a certain age I should be having a boyfriend, yeah.” (P13, S8, Teachers)*

### Sexual pleasure, judgement, and possible benefits

Another emerging perception although voiced in fewer FGDs (and not amongst any teacher FGDs) was that some girls chose to have TS because they enjoyed a sexual relationship and may thus be an initiator of a transactional sexual relationship.

*“Okay, this issue of girls forced to have sex, or willing to do sex, it depends with the girls also. You know there are some girls who are sexy. They just love that sex. They love it so much such that a day they visit their boyfriends, they know that day is for service. [Laughter] And if sex is not there she will feel bored. But there are girls who do not like it and they are forced. So that also depends with the girl.” (P13, S3, Girls)*

Whilst the tone of most FGDs were sympathetic or matter of fact in discussing the girls' behaviours, one of the schoolboys FGDs had some undertones of scorn or blame. The following excerpts from FGD S6, Boys illustrate:

“*Those monies they are given by the boda boda riders, most of them do not know how to use them, you will find someone just use it carelessly. She might go where the chapattis are sold, and use all the money to buy chapattis. She cannot think of saving some money for future use.” (P2, S6, Boys)*

*“If you want to know a girl is in need of a man, first of all, you should look at the dressing. You will find some of them wearing too short dresses. And when she wears a short dress, she has the intention of passing in front of you so that you can notice her. You know men are attracted to what they see.” (P2, S6, Boys)*

Only one participant across the FGDs noted that there may be some additional benefits in engaging in TS other than to directly obtain money, items or lifts. Although the chances are slim, according to this schoolgirl, she believed:

“*Some ’sponsors’ do pay girls school fees in return when you finish your schooling maybe he will marry you.” (P2, S5, Girls)*

## Consequences of transactional sex

### School dropout

Participants from all groups identified the role that TS had in leading girls to drop out of school. The schoolboys and girls FGDs perceived that the greater the engagement in TS, the greater the likelihood that she will drop out of school for various reasons including the perception that education becomes less important to the girl if she is able to have access to financial support.

“*What I can say is that, as long as that action is in place [engaging in TS], the more the chances of a girl staying in school is minimized. Because she will feel that this man with a motor bike; has money. So, what she will do is to follow money.” (P3, S6, Boys)*

*“Some thinks that the amount of money they get from “sponsors” is too much therefore no problem even if they drop, education will not help her, so she drops.” (P5, S5, Girls)*

### Pregnancy

The second reason stated related to risk of pregnancy, which then led to school drop-out. Although the role of TS causing pregnancy was only briefly discussed in the FGDs, many participants from all groups remarked on the hardships that came with schoolgirl pregnancies. School drop-out due to pregnancy occurs, according to the majority of adolescent girls, because (1) their schools believe pregnant schoolgirls to be a bad influence on their classmates or (2) their parents do not see a need to continue (paying for) their education once they have become pregnant. In both instinces, without parental or school support, the schoolgirl has little choice, but to drop out. Although this viewpoint was prevalent among the schoolgirls, only one teacher voiced a similar opinion. By law schools in Kenya are required to allow girls who are pregnant to continue their education, which may account for the lack of discussion surrounding this specific topic in the Teacher FGDs. It is also important to note that although the law may encourage girls to continue their education after delivering, but societally they may no longer be accepted as a schoolgirl and may be seen as not belonging in schools post delivery.

“*In other schools they do not allow that, when they know you are pregnant, they will just give you permanent suspension, you will not come back again.”* (P9, S5, Girls)

“*She will drop school because most parents when they see you pregnant, they will refuse to pay your school fees, they will tell you go to the person responsible for your pregnancy. So, it will force you to drop because your fees cannot be paid anymore. They say you never wanted education*.” (P1, S6, Girls)

“*So, you find that the boy child only runs the risks of contracting diseases but the girl child after experimenting with her body at that stage, which we do not have full control over, they stand a risk of may be getting pregnant, which will automatically force them to drop out of school.*” (P10, S2, Teachers)

Many schoolgirls remarked that once a girl becomes pregnant, she is often abandoned by the person responsible for the pregnancy and is left to deal with the repercussions alone.

“*If I can add, boys always love girls and that is true, he only loves you before you are pregnant. But once you are pregnant, you can never believe that boy was once yours. He will abandon and leave you.” (P5, S3, Girls)*

Despite TS being described in the narratives as the norm, once a girl becomes pregnant her behaviour becomes stigmatized. At that stage friends, family, community adults, and other peer groups use derogatory terms to describe her.

“*At home the parent will tell you, she will call you names, she will say, you are a prostitute, you are pregnant, and she does not want to see you in this home, you find somewhere to go, so you will feel your life is already ruined and you need to get married, or maybe take poison.”—(P5, S2, Girls)*

*“Stigma, they are seen as prostitutes by other girls, yeah that stigma.”—(P5, S2, Teachers)*

One girl observed that even after giving birth, the stigma associated with teen pregnancy continues into motherhood.

“*It will be different from the way it was before she got pregnant, you know now she is a mother, people call her mother, she will be ashamed being called ‘mother’.” (P2, S1, Girls)*

### STIs

STIs were also a risk associated with engaging in TS and were briefly discussed in the schoolgirl FDGs, although much less narrative was devoted to this in comparison to pregnancy. Several girls and one parent remarked that girls who engage in TS are unknowingly exposed to STIs and are at risk of infection.

“*Some of the boys have diseases like gonorrhoea, syphilis and you are not aware so when you have sex with them you will get infected.” (P1, S6, Girls)*

*“Some boys when you have sex with them, you will be infected if you do not use protection, and apart from that you can get pregnant, early pregnancy.” (P4, S8, Girls)*

*“Of course, the motor bike operators will want them to pay with their body. You have sexual intercourse with them and then get infected.” (P11, S2, Parents)*

## Discussion

This qualitative study adds to the expanding body of research exploring the complexities driving TS among AGYW in LMIC. Our study provides insights into the perspectives and experience of AGWY in rural Kenya, enriched by perceptions from groups who form their peer or support networks. The findings from a series of FGDs confirm that TS is viewed as a commonplace and normative behaviour among AGYW who attend school in rural western Kenya and suggest that while poverty is the underlying driver behind TS there are additional important contributors fuelling these behaviours. These insights may be useful in helping to address the health and gender issues surrounding TS and are discussed in more detail below.

TS is common phenomenon in SSA (6, 30). Similarly, within our study there was clear acknowledgement across all groups and type of participant that TS is a customary behaviour in AGYW and is a culturally accepted norm. Generally, boys, teachers and parents spoke about this as normal behaviour with little censure of girls who participated; they understood why girls needed to adopt this behaviour. Likewise, narratives from the girls' FGDs demonstrate TS as expected, acceptable and a somewhat routine means to obtain necessities. Historically, literature has highlighted that TS is predominantly driven by basic survival or subsistence needs (2). As Wamoyi and colleagues describe (30), sex becomes a “commodity to be purchased” (30). Only once survival needs are met, is there more likelihood of girls being influenced by additional factors such as status, peer pressure or receiving symbolic tokens of affection that then become the cultural norm in relationships. Our findings suggest that the need for basic necessities was the greatest driver of TS amongst AGYW, with minimal overlap of sex for status or for material expressions of love, although peer pressure was a recognised contributor.

Additionally, according to all participant groups, some parents seemingly put pressure on their daughters or wards outright to either engage in TS, or subliminally by expecting them to bring home monies or goods; this later point indicates that the driver of TS in this setting is primarily for economic need. We consider that this stems from the study being set in rural western Kenya which is an impoverished region where minimal income earning opportunities exist for AGYW (and indeed often for their families) to purchase essential items that may be required for their day-to-day living, or to assist in school attendance or activities. Evidence from studies in Zimbabwe (31) and Tanzania (10) similarly indicate AGYW actively used their sexuality as a pecuniary resource, predominantly entering relationships for economic reward.

Parents appeared to hold a dichotomy of opinions and thus differing behaviours around AGWY engagement of TS. Many spoke of their own struggles to prevent their daughters from undertaking such behaviours and spoke of daughters engaging in TS without their knowledge or approval. On the other hand, our participants referred to “other” parents who they condemned for actively or passively encouraging TS in their adolescent daughters and/or accepting the material rewards. This contradiction may arise from sampling or methodological issues. Parents who wanted to participate in our FGDs may have been the most concerned for their daughter's health and so have a more nurturing perspective. Equally, social desirability may have biased responses with parents wanting to distance themselves from being party to their daughters engagement in TS. However, in a context where poverty and deprivation are the standard, it is possible that the desire to prevent daughters from having to resort to TS is offset by the need for necessities and an acceptance that they, the parents, cannot provide the means to do so themselves. The contradiction in language may reflect an attempt to reconcile wants with needs, and to disengage with their own daughters' actual behaviours, portraying an idealistic rather than realistic situation. A similar situation was described by Wamoyi et al. (30) “where parents communicated something (e.g., abstinence) and tolerated the opposite (transactional sex)” (30). The authors noted that parents discussed TS in relation other people's children, but when asked about their own children's behaviour parents thought their own children were not active in this way.

Other issues that emerged as important to girls in determining their needs for resources and therefore driving TS, were communication and the nature of their relationship with parents or carers. Girls sometimes felt unable to ask the “responsible” adult in the family for monies to purchase items such as sanitary pads or underwear. To some extent this was related to the nature of the product, i.e., it being a personal item that cannot easily be spoken about. This is frequently described in the research literature on menstruation (32, 33), across cultures and continents (34). However, generational gaps, such as a girl being cared for by her grandparent, or a gender gap, e.g., a girl being cared for by her father, made communication of such needs particularly difficult. Difficulties in communication are also driven by strained relationships. In this and our current and previous Kenyan studies we have noted that girls regularly state that their parents are “harsh”, sometimes also describe very difficult domestic situations. This has also been stated by parents and teachers alike in the FGDs. This outcome may be rather typical of adolescent behaviours where young people growing up are asserting their independence and attempting to break free of parental pressures, clashing as a result (35).

We note that it was common to “blame” the boda boda driver as a key predator in TS, and indeed a number of studies have similarly cited the boda boda in this manner (36, 37). However, this may also stem, at least in part, from a socially conditioned response whereby boda boda have a reputation within the local community as predators. It is possible that boda boda are seen as predators as girls often need lifts (to school) so have little recourse but to pay with their bodies. In contrast, the older and wealthier partners may be more socially acceptable and “targeted” as partners by girls as they can offer better rewards. The latter phenomenon has been reported in several studies which suggest TS promotes age-disparate relationships as AGYW seek out older partners because of their greater wealth (38–40).

Also of major concern, and seemingly not an unusual occurrence were teachers as TS partners of students. In our narratives, girls, schoolboys and teachers themselves mentioned their role in having sexual relationships with pupils. This was spoken of in a matter-of-fact way, further echoing the seeming normality of the situation. In one of the teacher FGDs this topic was discussed in a very light-hearted way with much laughter and hilarity which suggests some teachers themselves are not shocked or condemn this type of relationship. Other studies have reported on the same phenomena (41–43). Whilst studies rightly suggest understanding of the context in which TS takes place is needed (13) surely there is no situation wherein a teacher can justify having a sexual relationship with a pupil irrespective of gender, age of pupil or paradigm setting.

Our previous study conducted in the same area was undertaken with girls attending primary school and it was found that some girls had TS though their discourse on the matter was qualitatively different. The girls took no ownership of engaging in TS and were keen to state that it was “others”, not themselves who acted in this way (22). In our present study it was interesting to discover that girls had no compunction in owning this act and did not reflect it back upon others. As both studies involved the same methods of data collection and the same moderator it is unlikely this was a methodological issue. We anticipate therefore the difference is largely explained by school setting i.e., primary vs. secondary participants determining the acceptability of behaviours as there was some age overlap. In Kenya it is grade completion rather than age *per se* that is the determinant between primary and secondary schooling. We included girls aged 14–16 in the primary school study and girls 15–18 in the present study. Our reflection is therefore that interventions pertaining to TS may be of more benefit targeting primary school girls in order to prevent the cycle of TS being an acceptable and normalised behaviour. This would also tie in with the need to introduce interventions at or around the time of menarche (see later).

Other studies (21, 22) have similarly documented menstrual pads as necessary items that girls purchase with proceeds from TS, or menstrual products acts as a motivator in engaging in TS for adolescent schoolgirls. The present study is however the first, to our knowledge, that documents the needs for menstrual pads as an actual trigger point for girls initiating TS. Whilst these are the perceptions only of a few participants, and so we do not want to overstate their critical role in the initiation of TS, cumulative evidence leads us to emphasise how important access to menstrual products are in contributing to girls TS behaviours (44, 45). Whilst currently there is not enough evidence to suggest that provision of products, (or provision of the means to access products) may prevent TS altogether, it is possible it may delay entry into a habit of engaging in TS or reduce the number of transactional acts. Each TS encounter with an age-disparate partner and without use of a condom (13, 46, 47) is a high risk for impregnation or infection. Given that the median age of menarche in our population was 15 years, with early menarche (<13 years) associated with adolescent pregnancy (23) it would seem important that timely interventions i.e., at an early age coinciding with onset of menarche, are needed to prevent high risk sexual and reproductive harms to vulnerable girls. Studies suggest additional benefits to schoolgirls from provision of or access to menstrual supplies include; greater participation in schooling, possible reduction in school absenteeism, greater confidence alongside reduction in menstrual related shame and stigma, greater personal comfort, fewer hygiene related issues such as odour and infections (22, 48). All of these reasons provide a strong argument that schoolgirls who lack access to suitable menstrual products need assistance as a matter of health and gender equity.

A striking anomaly emerging from the narratives was that whilst TS was seen as acceptable behaviour amongst AGYW any resulting pregnancy (including that from a non-TS encounter) left the girl stigmatised as a prostitute. Seemingly then TS is more culturally accepted in rural Western Kenya than adolescent pregnancy. Hypothesising why this conundrum may occur, we speculate if it is because TS is somewhat “invisible” *per se* although acknowledged in general, yet a pregnancy is obvious to the community at large. The pregnant girl is visibly seen to have brought shame and disgrace on her family, particularly if she does not (or cannot) marry the father. Instead of bringing some “sugar” to the family, she has brought another mouth to feed. Unsurprisingly, therefore, pregnancy was the primary fear spoken of among schoolgirls participants in all FGDs, with few mentioning HIV/STI's as a possible risk from engaging in TS. They acknowledged the stigma associated with pregnancy and how falling pregnant while still in school could negatively impact their relationships and life trajectory. Multiple girls stated those who became pregnant were often abandoned by their partner and forced to seek help from family who may refuse to allow her to stay at home. Other studies have similarly found that in age-disparate transactional sexual relationships, adolescent girls are much more concerned about pregnancy and/or loss of monetary support than any HIV or STI risk (49).

We acknowledge some limitations in our study. We noted that male participants spoke from the perspective of a parent, a teacher or a schoolboy and peer to the girls, yet it is quite possible that they also had another persona, that of a transactional partner. However, this positionality did not emerge during the discussions. This may have resulted from our sampling, bias towards social desirability response, the research invite which identified them as a “parent”, “teacher”, or “schoolboy peer”, or the framing of the questions which echoed those same categories as above rather than as a potential transactional partner. Had we sought their viewpoints as potential transactional partners, this would have enriched our data and provided a more rounded view of the phenomena of TS in this population.

A second limitation of the study is the sole use of FGDs to gather data. These may reflect social desirability response to “appease” the moderator and note-taker, or to agree with the perspectives of the more vocal participants. We attempted to limit the former by having a moderator and note-taker who were local and reside in the study area, and who are very experienced in conducting FGDs within the community. We believe that the similar experiences and perspectives being echoed throughout and across the FGDs suggest the data does largely reflect the prevailing attitudes of the participants.

Finally, the resulting paper arose from a deductive rather than an inductive approach as we focused specifically on the emerging findings around transactional experiences rather than a wide exploratory approach that our original study research questions sought. Our analysis was not strictly a like by like comparison across groups and so it is inevitable that there will be differences in codes which would not necessarily determine the importance of one code above another, either between or across the various groups. We would argue that the analysis has reduced the coding to a meaningful comparison and contrast within and across the groups. This gives the reader a snapshot of the context and the key findings.

## Conclusion

Our study suggests that TS is prevalent among secondary schoolgirls in rural western Kenya and accepted as the norm. The key driver appears to be poverty. Parents cannot provide many of the basic necessities for their daughters who resort to TS as a means to obtain, whether for their own individual needs or those of their family. This suggests that although TS is seen as acceptable it is not really a choice. Therefore, solutions are needed to reduce adolescent schoolgirls risk; and also to consider mechanisms to mitigate the physical, psychological and emotional harms arising from it. Further, exploring ways to encourage communication between families, including around menstruation, may help enable girls to ask for help in purchasing their requirements. In addition, education at a community level may shift social norms overtime and decrease the prevalence of age-disparate transactional sex among AGYW and older men in the community.

## Data Availability

The datasets presented in this article are not readily available because This study was conducted with approval from the Kenya Medical Research Institute (KEMRI) Scientific and Ethics Review Unit (SERU), which requires that data be released from any KEMRI-based Kenyan studies (including de-identified data) only after their written approval for additional analyses. In accordance, data for this study will be available upon request, after obtaining written approval for the proposed analysis from the KEMRI SERU. Their application forms and guidelines can be accessed at https://www.kemri.org/seru-overview. Requests to access the datasets should be directed to seru@kemri.org.
